# A Brief Review of Higher Dietary Protein Diets in Weight Loss: A Focus on Athletes

**DOI:** 10.1007/s40279-014-0254-y

**Published:** 2014-10-30

**Authors:** Stuart M. Phillips

**Affiliations:** Department of Kinesiology, Exercise Metabolism Research Group, McMaster University, 1280 Main St. West, Hamilton, ON L8S 4K1 Canada

## Abstract

Thermodynamics dictates that for body weight (i.e. stored substrate) loss to occur a person must ingest less energy than they expend. Athletes, who owing to their oftentimes large daily energy expenditures, may have greater flexibility than non-athletes in this regard; however, they may also have different goals for weight loss. In particular, weight lost may be less important to an athlete than from which compartment the weight is lost: fat or lean. A critical question is thus, what balance of macronutrients might promote a greater fat loss, a relative retention of lean mass, and still allow athletic performance to remain uncompromised? It is the central thesis of this review that dietary protein should be a nutrient around which changes in macronutrient composition should be framed. The requirement for protein to sustain lean mass increases while in negative energy balance and protein, as macronutrient, may have advantages with respect to satiety during energy balance, and it may allow greater fat loss during a negative energy balance. However, athletes should be mindful of the fact that increasing dietary protein intake while in negative energy balance would come at the ‘expense’ of another macronutrient. Most recently there has been interest in lower carbohydrate diets, which may not allow performance to be sustained given the importance of dietary carbohydrate in high-intensity exercise. The relative merits of higher protein diets for athletes are discussed.

## Introduction

Requirements for protein for the general population are defined by various agencies but generally appear in the range of 0.8–0.9 g protein/kg/day. In Canada and the US the recommended dietary allowance (RDA) defines the RDA as “… the average daily intake level that is sufficient to meet the nutrient requirement of nearly all [98 %] healthy individuals …”. The panel also states that “… no additional dietary protein is suggested for healthy adults undertaking resistance or endurance exercise” [1, 2]. It may be true that the basal ‘requirement’ for protein, even for the most intensely training athlete, is satisfied by the protein RDA. That is, 0.8 g protein/kg/day can satisfy the needs for all amino acid-requiring processes and that most athletes could likely even achieve nitrogen balance when consuming this intake; however, a pertinent question is whether such a state would result due to some adaptive change in an amino acid-requiring process and whether this adaptive change would compromise some goal in an athlete? Nevertheless, this is not a question that is easy to answer as a number of reviews [3, 4] and position stands [5] have concluded higher protein is required for athletes. The problem then of the discrepancy between population estimates of protein requirements [1, 2] and position stands on protein requirements for athletes [3–5] is more than likely that minimal intakes of protein can sustain normal function for the general population, but athletes are trying to optimize their adaptation to training. Thus, protein ‘requirements’ is the wrong term to use when referring to an athlete, and a more precise term to use is that of defining an optimal protein intake for an athletic population versus a protein intake to achieve nitrogen balance [1, 2]. This sentiment may be particularly true during an energy deficit when the choice of which macronutrients to consume may be even more critical, at least from an athlete’s perspective.

A thermodynamic reality of weight loss in humans (i.e. a net oxidation of stored substrates) is that total ingested energy needs to be less than total energy expenditure over some defined period of time. The result is a net loss of body weight that is usually comprised, from a tissue standpoint, of stored lipid and lean tissues in a ratio of about 3:1 [6]. A more rapid weight loss can shift this ratio toward greater lean tissue loss [7] even in athletes [8]. From an athlete’s standpoint it may be more important to favor weight loss that emphasizes fat loss and muscle preservation, which may be more conducive to preservation or increases in performance. In addition, in a number of sports it is generally recognized that a high strength, power, or endurance to body weight ratio is desirable. We have referred previously to weight loss that has a high fat:lean ratio as higher quality weight loss [9]. In fact, in certain circumstances it is desirable for athletes to increase their lean mass while losing weight [10].

The aim of this article is to provide a brief review of the evidence examining why protein might be considered the macronutrient around which to base a hypocaloric weight loss diet owing to its role in satiety, thermogenesis, maintenance of lean mass, and utility in supporting adaptation to training.

## A Different Approach to Determining Optimal Protein Intakes

We had previously reported that in ~87 kg males, a dose of egg protein that maximally stimulated muscle protein synthesis was 20 g [11]. Recently, Witard and colleagues [12] confirmed, using whey protein and in the fed state, that the same dose of protein was sufficient to maximally stimulate muscle protein synthesis. Thus, despite the capacity to be able to digest more protein, there is obviously a finite capacity to put amino acids into skeletal muscle. Indeed a ‘muscle full’ phenomenon has been described following meal ingestion [13]. Importantly, however, is what the protein dose per meal might be on a body weight basis to allow adjustment for smaller or larger athletes. Estimations based on the data we have at present [11, 12] are that a per-meal ‘dose’ of protein of ~0.25 g protein/kg would optimally stimulate protein synthesis [14]. With this per meal ‘dose’ in mind, one can begin to formulate a protein consumption strategy based around periodic stimulation of protein synthesis, which is in fact what was trialed by Areta et al. [15]. In this investigation, a group of young men who had just performed resistance exercise had the largest stimulation of muscle protein synthesis, with protein ingestion of 20 g (~0.25 g/kg) every 4 h versus 10 g (~0.12 g/kg) every 2 h or 40 g (~0.48 g/kg) every 8 h [15]. These findings provide at least a proof of principle that a per meal protein dose of ~0.25 g protein/kg/meal seems to be optimally effective, at least in stimulating muscle protein synthesis. Indeed, we have recently confirmed this dose does represent an optimally effective dose of protein for young men [14]. While larger protein doses can definitely be digested, they appear not be able to further stimulate muscle protein synthesis but do lead to marked amino acid oxidation [11] and urea synthesis [12]. One important consideration in interpreting the results from acute feeding trials [15] is that they represent an acute response to protein-only feeding, and the influence of other nutrients and energy balance are unknown. Also, the long-term translation of acute findings to chronic phenotypic changes requires caution in interpretation. Nonetheless, if we accept that a per meal dose of 0.25 g protein/kg/meal is a reasonable estimate and means of defining an optimal protein intake then this could allow the calculation of daily recommendation for an athlete looking for optimal protein intake. Using this approach and including four discreet eating occasions per day as well as one pre-sleep meal that is twice as large (i.e. 0.5 g protein/kg/meal) to offset catabolic losses during sleeping [16], then a 100 kg athlete would be consuming four meals of 25 g of protein plus one meal of 50 g of protein for 150 g of total daily protein or 1.5 g/kg/day. One could argue that more eating occasions could be required but it appears that such a feeding pattern would result in a relatively sustained daily hyperaminoacidemia, which has been shown to result in a refractory response of muscle protein synthesis [17].

## Protein as Centrally Important Macronutrient in Weight Loss

Consumption of protein at higher-than-recommended levels has been theorized to have a number of potential advantages during weight loss, including a greater thermogenic effect upon consumption compared with carbohydrate and fat [18], a greater satiety response on consumption [19, 20], and the potential for greater weight loss, fat loss, and lean mass retention [21, 22]. In addition, it has been proposed that protein could actually reduce, presumably through the independent and/or synergistic effects outlined above, the intake of other nutrients due to a homeostatic mechanism based around a protein ‘seeking’ behavior termed by Simpson and Raubenheimer [23] as the protein leverage hypothesis. Trials of this hypothesis [23] have been undertaken and, in general, protein intakes lower than 10–15 % of energy are associated with greater daily energy intake than those above these levels [24, 25]. In addition, a recent meta-analytical study of ad libitum energy intakes provides unique evidence that non-protein (i.e. fat and carbohydrate) energy intake increases with declines in percent dietary protein [26]. The evidence was more convincing for intakes below 20 % of total energy as protein as a bona fide driver of energy intake than intakes of energy above this level [26]. If the protein leverage hypothesis is correct, and for many of the reasons outlined above, there is reason to promote higher protein intakes during an energy deficit.

One consideration for athletes wishing to lose weight is that if they undertake a hypocaloric diet and, as recommended, increase their protein intake, then another macronutrient intake would have to be reduced. While there are many who propose a higher fat (and presumably higher protein), lower carbohydrate diet, such a diet has not been shown to be effective in allowing exercise performance at the higher exercise intensities [27, 28]. Thus, it would be prudent for athletes who are aiming to sustain/improve their training intensity that it be lipid energy that is sacrificed in an energy deficit and that protein and carbohydrate are emphasized. While it is beyond the scope of this review, recent guidelines for carbohydrate are given and based on levels of exercise volume and intensity [29, 30]. However, despite these recommendations, athletes who are not engaging in high-intensity training, or are in a period of their training cycle in which intensities and volume are low, could maintain adequate training and performance with lower carbohydrate intakes. Such a low carbohydrate strategy may be advantageous in a weight-loss situation as lower carbohydrate and higher protein intakes are associated with greater weight loss, greater fat loss, and retention of lean mass [21, 22], at least in obese non-athletic individuals. However, it is possible that athletes can modify their body composition while training through well-planned consumption of macronutrients, still emphasizing protein to retain lean mass while maintaining their training [10].

## Protein Intakes in Hypocaloric Situations: What Level?

Meta-analysis [21] and meta-regression [22] in non-athletes have shown that compared with normal protein intakes (i.e. 12–15 % of energy intake from protein), higher protein intakes (25–35 % of energy intake from protein) can attenuate the hypocaloric-induced reduction in skeletal muscle mass and also promote greater reductions of fat and total body mass. In addition, resistance exercise is also a potent stimulator of muscle protein synthesis to the extent that it too can result in greater net retention of lean mass during an energy deficit [6, 31]. In fact, a higher protein diet combined with exercise (in some cases resistance, and in other cases a combination of aerobic and resistance) has been shown to result in a relative sparing of lean mass [8, 9, 32, 33]. A recent systematic review in resistance-trained athletes by Helms et al. [34] examined what protein intakes might offset weight loss in ‘lean’ athletes in various hypocaloric situations. Based on examination of only 13 studies, it was observed that in nine of these studies lean (i.e. fat-free) mass was retained or increased. However, problems with many of the studies examined included the substantial heterogeneity of study design, the high degree of variability in protein dose or the complete lack of a high and low protein dose [35], failure to control the training of the subjects during the hypocaloric period, plus differential times spent losing weight (allowing for more training in one group) [8], and small sample sizes [32, 35, 36].

Nonetheless, despite the limitations of the studies examined, the authors concluded that higher protein intakes 2.3–3.1 g/kg/day of protein was required to offset losses of lean mass [34]. Others, based on collective examination of data, have also hypothesized that much higher protein intakes are required to see preservation of lean mass and greater fat mass losses [37]. In contrast, a recent study by Pasiakos et al. [38] found that lean mass retention tended to be greater in a group consuming 1.6 g/kg/day versus a group consuming 2.4 g/kg/day. Thus, it would seem spurious at the present time to make specific recommendations about an exact protein dose. Suffice to say, however, that the sum of available evidence indicates that protein intakes higher than the RDA (1.3–1.8 g/kg/day) [39], possibly substantially greater (2.3–3.1 g/kg/day) as some have recommended [34], can offset lean mass losses. However, factors influencing specific recommendations would have to take into account the training status, goals, rate of weight loss (i.e. energy deficit), and training volume during the hypocaloric period.

## Higher Protein Diets and Renal Health

An often-cited potential problem with higher protein diets is the potential risk such diets may pose for renal health. It is likely that these comments are made in light of the knowledge that people in renal failure benefit from protein-restricted diets [40]. Notwithstanding this evidence [40], a circular argument regarding higher protein and renal health in people with normal renal function cannot be made; that is, because people with poor renal function benefit from a lower protein intake does not mean that athletes with normal renal function who consume high protein will have problems with their renal health [41, 42]. In fact, an examination of the statements made by both the Institute of Medicine in setting the protein RDA in North America [1], as well as the World Health Organization’s (WHO) report on protein intakes [2], indicates there is no evidence linking a higher protein diet to renal disease. As the WHO report [2] states, “… the suggestion that the decline of glomerular filtration rate that occurs … in healthy subjects … can be attenuated by reducing the protein in the diet appears to have no foundation”.

In agreement with the WHO’s conclusion, the panel setting the Australian and New Zealand Nutrient Reference Values [43] concluded “There is no published evidence that a diet containing up to 2.8 g protein/kg/day produces adverse effects on kidney metabolism in athletes. In addition, no known association of protein intake with progressive renal insufficiency has been determined [44]”.

## Conclusions

Dietary protein is a critical macronutrient for athletes and is required on a daily basis. While the current RDA likely provides sufficient protein for athletic performance, it may not provide an optimal level of dietary protein to allow for adaptation. Current evidence appears to support a per meal recommendation of 0.25 g protein/kg/meal [14], with a larger meal prior to sleep (see Sect. 2.0). Based on available evidence, protein appears to be the macronutrient of paramount importance during weight loss owing to its ability to preserve lean mass during weight loss and promote fat mass loss when consumed in higher quantities. In addition, protein has some notable characteristics as a macronutrient, including satiety, thermogenic effect, and a potential ‘leveraging’ effect that means it should be a central part of a plan to restrict dietary energy to promote weight loss. While a specific recommendation on exactly how much protein should be consumed is lacking, estimates have ranged from intakes between 1–3 and 1.8 g protein/kg/day to much higher. While higher protein intakes, particularly in their ability to spare lean mass during energy restriction, have been speculated to be effective, there is scant data to support recommendations for very high protein intakes (i.e. >2.5 g/kg/day) at the present time since they offer no apparent body composition or performance benefit. It may be that athletes also need to balance the increase in protein consumption with what macronutrient is reduced. The prudent advice for athletes would be to focus on reducing intakes of lipids to allow carbohydrate intakes to achieve performance. Finally, despite a widespread belief that higher protein diets will somehow compromise renal function, no such evidence exists to support this belief.
